# Comparative efficacy of locally isolated fungal strains for Pb(II) removal and recovery from water

**DOI:** 10.1186/s13065-017-0363-4

**Published:** 2017-12-20

**Authors:** Kiran Aftab, Kalsoom Akhtar, Razia Noreen, Faiza Nazir, Umme Kalsoom

**Affiliations:** 1 Department of Chemistry, Government College Women University Faisalabad, P.O. Box 38000, Shafique Road, Madina Town, Faisalabad, Pakistan; 2 Industrial Biotechnology Division, National Institute for Biotechnology and Genetic Engineering (NIBGE), P.O. Box 577, Jhang Road, Faisalabad, Pakistan; 3 Department of Applied Chemistry and Biochemistry, Government College University, Faisalabad, Pakistan

**Keywords:** Pb(II), Cyclic studies, Thermodynamics, Kinetics, Equilibrium study, Regeneration

## Abstract

The present investigation aimed to study and compare the efficiency of non-viable fungal isolates to remove divalent lead (Pb(II)) from aqueous streams. The selected fungal isolates showed identity with *Aspergillus caespitosus*, *Aureobasidium* sp. RBSS-303 and *Aspergillus flavus* HF5 as confirmed using gene sequencing of ITS regions of the ribosomal DNA (rDNA). The obtained equilibrium data for Pb(II) biosorption of *A*. *caespitosus* fitted better to Langmuir isotherm with maximum sorption capacity of 351.0 mg/g and *A.* sp. RBSS-303 and *A. flavus* HF5 showed good fit to Freundlich isotherm with maximum sorption capacity of 271.5 and 346.3 mg/g respectively. The values of thermodynamic factors ascertained the nature of adsorption process is endothermic with *A. caespitosus* and *A. flavus* HF5 but exothermic with *A.* sp. RBSS-303. The experimental data for Pb(II) biosorption fits very well to pseudo second order kinetic model. With HCl the maximum 85.5, 75.3, 73.7% recovery of Pb(II) was obtained from *A. caespitosus, A.* sp. RBSS-303 and *A. flavus* HF5, respectively. The observed percentage loss in sorption capacity of Pb(II) was 3.9% by *A. flavus* HF5, 12.2% by *A. caespitosus* and 26.6% by *A.* sp. RBSS-303 after five cyclic studies of sorption and desorption. Results from the study confirmed the efficiency order of *A. caespitosus* > *A. flavus* HF5 > *A.* sp. RBSS-303 to remove and recover Pb(II) from aqueous solution. Finally, the fungal biosorbents can be used as soil conditioning agent after compositing into valuables fungal protein.

## Introduction

A massive amount of toxic contaminants is discharged into water bodies and has become a severe hazard to environment with accumulation of non-biodegradable toxins in food chain [1]. Among different hazardous substances, lead Pb(II) is included in most toxic group of metals that form complexes by binding with negatively-charged organic molecule [2]. Different levels and concentrations of Pb(II) accretion cause various kinds of biological disorders in human body. Velmurugan et al. (2014) [3] reported that in UK the adults consume 1.6, 20, 28 μg of Pb(II) concentrations daily from air, water and food, respectively. At the same time, according to global non-renewable natural resource analysis (2000–2008) lead (Pb) reserves in nature are becoming scarce with the increased production rate of 1.5–2.2%, which showed the way to the need of simple and greener exclusion from the wastewater and its revitalization.

The increasing awareness of environmental issues laid down restrictive legal standards for maximum satisfactory concentrations of discharged metal ions in water and soil [2]. In developing countries, the water pollution situation is more alarming due to technology and management constraint. In Pakistan, only 1% of wastewater from industries is treated before being discharged [4]. Therefore, treatment and revitalization of resources from waste water stream have become a critical research topic for efficient and practical solution of water pollution [5]. Different adapted physical and chemical engineering technologies to treat the industrial effluents are commercially unfeasible due to high operational cost, creating other disposal problem, furthermore, also become inadequate to treat low concentrations (100 mg/dm^3^ or below) of metal ions. Therefore, the exploration of new most favorable technology to switch the conventional methods is the need to get rid of noxious waste to the lower point of requisite by regulation [6]. A multidisciplinary technique (biosorption) that comes with metal removal and recovery processes has focused in current research because of high competence in detoxifying dilute effluents, minimization of disposable chemical/biological sludge volume and low down operating cost [7]. Among various biosorbents (bacteria, fungi, algae, industrial and agricultural wastes, natural residues, other chitosan and cellulose-driven materials) fungi are attractive choice due to massive and easy growth with economical substrate in wastewater treatment processes [7–10]. Other biomaterials produce waste sludge heaps that increase the cost of effluent treatment plant. Fungi notably decrease the effluents treatment plant cost to alter the wastewater organic substance into valuables fungal protein (source of animal feed), also more effectively metabolize complex carbohydrates into large range of enzymes and biochemical’s [11, 12].

Therefore, to obtain maximum removal and recovery of Pb(II) from aqueous solution using indigenously isolated fungal strains, the present research was planned as following

- Screening of various fungal cultures for their Pb(II) binding potential.
- Identification of screened fungal isolates.
- Optimization of different experimental conditions for bio-removal of Pb(II).
- Analysis of biosorption data using different equilibrium, kinetic and thermodynamic models.
- Desorption studies of loaded biomass for recovery of sorbed Pb(II) using different desorbents.
- Cyclic sorption–desorption studies to evaluate the repeated use of biomass.

## Material and method

### Fungal culture growth conditions

Twenty-five fungal cultures were obtained from Laboratories of Industrial Biotechnology Division, National Institute for Biotechnology and Genetic Engineering (NIBGE), Faisalabad. These locally isolated fungal strains were screened for Pb(II) biosorption capacity. Vogel’s media was used to revive the biomass at 180 rpm, 28 ± 2 °C and harvested after 72 h.

### Identification and evaluation of fungal isolates

The selected fungal strains were initially identified on the basis of macro- and microscopic characteristics and further confirmed using molecular approach. For molecular typing of fungal isolates, the total genomic DNA of fungal isolates was extracted by cetyl trimethyl ammonium bromide (CTAB) method [13] that was partially modified in our lab as per requirements. DNA samples were used to amplify internal transcribed spacer (ITS) regions through PCR using universal primers ITS1 (Forward Primer): TCC GTA GGT GAA CCT GCG G and ITS4 (Reverse Primer): TCC TCC GCT TAT TGA TAT GC [14]. The PCR followed conditions were; 94 °C for 3 min, 94 °C for 30 s, 56 °C for 1 min 30 Cycles, 72 °C for 1 min, 72 °C for 10 min [14].

The amplified ITS regions/18S rRNA genes of isolates were partially sequenced commercially (Macrogen, Korea). These sequences were compared with other sequences of fungi present in the GenBank databases using the NCBI BLAST tool (http://www.ncbi.nlm.nih.gov) and then aligned with them using CLUSTALX [15]. The aligned sequences were used to construct a distance matrix, after the generation of 100 bootstrap sets that was subsequently used to construct a phylogenetic tree, by neighbor-joining method, using TREECON software. The partial ITS/18S rRNA gene sequences of theses isolates were submitted to GenBank to get Accession Numbers.

### Metal solutions

The lead (Pb) stock solution (1 g/dm^3^) was prepared by dissolving Pb(NO_3_)_2_ in distilled water. The different working solution concentrations were prepared by diluting the stock solution with distilled water. All other reagents used in the present research were of analytical grade (BDH, Sigma-Aldrich or Biolab brands).

### Batch biosorption trial

Batch biosorption trials were studied through shake flask method by adding known amount of biosorbent to Pb(II) solution (100 mL) of known concentration. The pH of the solutions was adjusted at 4.5 by dil. HNO_3_/NaOH before experiments.

The optimization of various factors like biosorbent culture (24–144 h), pulp density (0.1–0.75 g/L) and initial Pb(II) concentration (100–600 mg/dm^3^) was studied in a series of experiments. The experimental setup without adding the biomass serves as control. Time course studies were carried out to collect the 1.0 mL sample after specified time intervals. The collected samples were centrifuged at 10,000 rpm for 5 min and cells were discarded. Supernatants were exploited to determine the remaining Pb(II) concentration in solution on atomic absorption spectrophotometer (Model Varian 240 FS). The biosorbed amount of Pb(II) mg/g dry weight of biomass (q) is called biosorption capacity and was calculated using following concentration difference method:

1 $${\text{q }} = \, \left( {C_{\text{i}} - C_{\text{f}} } \right){\text{V}}/{\text{ W}}$$

where *C*_i_ and *C*_f_ (mg/L) are initial and final Pb(II) ion concentration in aqueous phase, W (g) is the weight of suspended biosorbent and V (dm^3^) stands for volume of Pb(II) solution.

### Metal elution and regeneration of biosorbents

Different desorbents [distilled water, HCl, CH_3_COONa, NaOH, Na_2_CO_3_, NaHCO_3_, (NH_4_)_2_SO_4_ and (NH_4_)_2_CO_3_] were screened to recover the accumulated Pb(II) and to regenerate the exhausted biosorbents. Pb(II) loaded biomass (0.025 g) was washed with distilled water and mixed in 30 mL of 0.01 M desorbents at 180 rpm and 28 ± 2 °C. Regenerated biomass was washed, filtered and finally dried at 60 °C to calculate the loss in weight. The regenerated biomass was used for next sorption experiment and this sorption desorption cyclic studies were carried out for five times. The value of eluted Pb(II) per gram of biomass (q_des_) was calculated from desorbed Pb(II) concentration (C_des_) as follows:

2 $${\text{q}}_{\text{des}} {\text{ = C}}_{\text{des}} {\text{V}}/{\text{W}}$$

where W (g) shows the biomass weight in V (dm^3^) volume of solution. The percentage desorption of Pb(II) was calculated as

3 $${\text{Percentage desorption = }}\left( {{\text{q}}_{\text{des}} /{\text{ q}}} \right) \, \times 100$$

The entire experimentation was carried out in three replicates and the obtained data was computed on Slide Write Plus 7.01 (Advanced Graphics Software Inc., Ranco Santa Fe, CA, USA). Mean standard deviation and correlation coefficient (r^2^) values were calculated according to standard equations.

### Equilibrium and kinetic modelling

Experimental data and biosorbent capacity was evaluated to use isothermal studies using Langmuir, Freundlich and Dubinin–Raduskevich adsorption models. The use of sorption isotherms and thermodynamic factors were pertaining approach to assess the feasibility of research work. The Langmuir model [16] describes monolayer sorption of adsorbed molecules on a homogenous surface without any interaction. This model can be written as

4 $$1/{\text{q}}_{\text{e}} = 1/{\text{q}}_{ \hbox{max} } + \, \left( { 1/{\text{q}}_{ \hbox{max} } \cdot {\text{K}}_{\text{L}} } \right) 1/{\text{C}}_{\text{e}}$$

where q_e_ is the adsorbed Pb(II) on biosorbent at equilibrium, C_e_ is the equilibrium Pb(II) concentration in the solution, q_max_ is the maximum biosorption capacity of biosorbent and K_L_ is the Langmuir constant involving the free energy of the process. The plot of 1/q_e_ versus 1/C_e_ gives a straight line having slope 1/q_max_ K_L_ and intercept 1/q_max_.

Freundlich model is another widely used isotherm, proposes a multilayer sorption of adsorbate on adsorbent active sites of heterogenous energy [17]. The Freundlich model is

5 $${\text{ln q}}_{\text{e}} = {\text{ ln K}}_{\text{F}} + 1/{\text{n ln C}}_{\text{e}}$$

The plot of ln q_e_ versus ln C_e_ generate Freundlich constants K_F_ (g^−1^), n is the biosorption extent. According to the Freundlich, the maximum adsorption capacity can be calculated from the following equation [18].

6 $${\text{K}}_{\text{F}} = {\text{ q}}_{ \hbox{max} } / {\text{C}}_{\text{i}}^{{ 1/{\text{n}}}}$$

Dubinin and Radushkevich (D–R) isotherm describe the effect of porous nature of biosorbent

7 $${\text{ln q}}_{\text{e}} = {\text{ ln q}}_{\text{m}} - {\ss}\varepsilon^{ 2}$$

By plotting ln q_e_ against ε^2^ (Polanyi potential = RT ln(1 + (1/C_e_)), the value of q_max_ (mole/g) and ß (mole^2^/(J^2^)) [19]. The constant ß give an idea about the mean free energy (kJ/mole).

The experimental data pertain to time course studies was subjected pseudo-second-order, saturation mixed order and intraparticle diffusion models. The model predicted values were validated by correlation coefficient values and with comparison of theoretical value with experimental one.

### Scanning electron micrograph–energy-dispersive X-rays analysis (SEM–EDXA)

To look at the Pb(II) sorption mechanism, scanning electron micrograph with energy-dispersive X-rays analysis (SEM–EDXA) of loaded and unloaded biomass was studied using

Scanning electron microscope equipped with EDX analyzer [20]. The samples were oven dried at 90 °C, grinded and immersed in little distilled water followed by vortex for 3 min. A small amount of samples were shifted to stub using a pipette then vacuum dried for 2 h at 80 °C. These samples were gold coated in sputter coater (10^−4^ pa) to make them good conductors, and images were collected by putting in SEM holder.

## Results and discussion

### Screening studies

The batch screening studies were conducted at initial pH of 4.5 by incubating freshly harvested wet biomass equivalent to 0.05 g dry weight in 100 mL of Pb(II) solution having 100 mg/dm^3^ initial concentration. The sorption capacity (q mg/g) of different isolates for Pb(II) biosorption varied from 11.6 ± 3.4 to 164.5 ± 3.5 (Table 1). After screening and rescreening for Pb(II) biosorption, three isolates BC01, BC04 and BC22 were selected for further identification and optimization of various factors that affect the biosorption process.

**Table 1:** Screening studies for Pb(II) biosorption

Biosorbents	Sorption capacity (q) (mg/g)	Biosorbents	Sorption capacity (q) (mg/g)
BC01	164.5 ± 3.5	BC14	23.4 ± 2.4
BC02	11.6 ± 3.4	BC15	20.2 ± 5.7
BC03	54.3 ± 2.5	BC16	28.7 ± 0.9
BC04	148.2 ± 5.2	BC17	24.9 ± 3.6
BC05	12.3 ± 4.3	BC18	33.5 ± 2.7
BC06	40.1 ± 3.3	BC19	61.6 ± 2.5
BC07	41.1 ± 1.8	BC20	40.2 ± 6.1
BC08	62.9 ± 1.3	BC21	58.7 ± 2.6
BC09	14.9 ± 2.6	BC22	71.4 ± 2.4
BC10	34.0 ± 2.0	BC23	29.7 ± 4.9
BC11	59.3 ± 2.2	BC24	65.2 ± 3.0
BC12	63.4 ± 3.4	BC25	49.7 ± 1.6
BC13	58.7 ± 3.6		

Wet weights equivalent to 0.05 g dry weights in 100 mL of 100 mg/dm^3^ Pb(II) solution

### Identification of selected isolates

The selected fungal isolates were first identified on morphological basis, and then reconfirmed by amplifying ITS sequences of these isolates using ITS 1 and ITS 4. The single pattern band was observed around 524–626 bp. The BLAST outcomes through the Gene Bank proved the regions of similarity of these local isolates with that available in database (Table 2). The fungal isolates BC01 and BC04 were found to be closely related to *Aspergillus* sp. exhibiting similarity values 98 and 99% respectively. However, isolates BC22 was found to be related to *Aureobasidium* sp. RBSS-303 with similarity value of 99%.

**Table 2:** Closest matches from BLAST searches of fungal sequences

Isolates	Closest species match (accession code)	Sequence identity (%)	Overlap sequence (bp)	References
BC01	*Aspergillus caespitosus* (AB267813.1)	98	902	[21]
	*Aspergillus caespitosus* (AY373841.1)	98	822	[22]
BC04	*Aspergillus flavus* HF5 (GU183169.1)	99	946	[23]
	*Aspergillus flavus* (GU183163.1)	99	959	[23]
BC22	*A*u*reobasidium* sp. RBSS-303 (GQ911532.1)	98	776	[24]
	*Aureobasidium pullulans* (FN428912.1)	99	809	[25]

### Optimization of physical and environmental parameters

#### Effect of physical state and culture age on biosorption capacities

For assessment of culture age and biomass physical state (wet/dry), each biosorbent (0.05 g dry weights/wet weight corresponding to 0.05 g dry weight) was incubated with 100 mL solution [100 mg Pb(II)/dm^3^] at 180 rpm, pH 4.5 and 28 ± 2 °C. All three selected fungal isolates with dry biomass (deactivated at 80 °C) gave high Pb(II) biosorption capacities as compared to viable, non-metabolising wet biomass. Pb(II) biosorption capacities were 164.5 ± 3.5, 65.2 ± 3.0, 148.2 ± 5.20 mg/g for viable and 174.2 ± 4.4, 79.9 ± 2.3, 160.7 ± 1.3 mg/g for non-viable *A*. *caespitosus, A.* sp. RBSS.303 and *A. flavus* HF5 respectively. Zouboulis et al. [26] also reported higher uptake of Cd(II) by dry biomass of *Bacillus laterosporus* and *Bacillus licheniformis* in contrast to that of viable biomass. The high efficiency of dry biomass for metal biosorption may be due to the fact that potential binding sites from intracellular components become exposed after heating, cutting and grinding. Keeping in view the ease of handling and high uptake capacity the dry biomass of respective biosorbents were used for further Pb(II) biosorption studies compared to that of wet one.

**Fig. 1:**
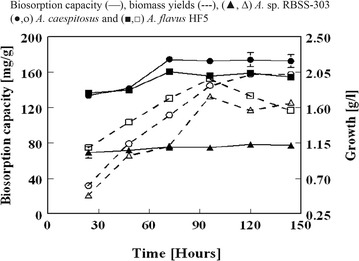
Effect of *A.* sp. RBSS-303, *A. caespitosus* and *A. flavus* HF5 culture age on Pb(II) uptake capacities (W = 0.05 g, pH = 4.5, temperature = 28 ± 2 °C, C_i_ = 100 mL, V = 100 mL, agitation rate = 180 rpm)

To observe the effect of fungal culture age on biosorption capacity, biomass was harvested subsequent to 24, 48, 72, 96, 120, and 144 h. Each one of these biomass was incubated at 28 ± 2 °C with shaking at 180 rpm employing 100 mL of 100 mg/dm^3^ Pb(II) solution. Significant difference in sorption capacities relative to harvesting time was observed for all the three biosorbents studied (Fig. 1). For *A.* sp RBSS-303 and *A. flavus* HF5, the maximum values of biomass yields were found to be 1.74 and 1.96 g dry weight/dm^3^ respectively on harvesting the biosorbent after 96 h of incubation. After 96 h, the biomass yield continued to decrease till 144 h of incubation. The maximum value of biomass yields observed was 2.02 g dry weight/dm^3^ for *A*. *caespitosus* after 120–144 h of incubation (Fig. 1). The highest observed values of Pb(II) accumulation on *A*. *caespitosus* and *A. flavus* HF5 were 174.4 ± 4.4 and 160.7 ± 1.3 mg/g dry weight using biomass harvested after 72 h and it was slightly decreased to 172.5 ± 5.9 and 154.4 ± 1.5 mg/g dry weight respectively when biomass harvested after 144 h was used. For *A.* sp RBSS-303, insignificant difference was found in uptake value using biomass harvested after 24–144 h of incubation and the maximum sorption capacity was found to be 79.3 ± 0.25 mg/g dry weight. With culture age, variation in sorption capacity may be due to the alteration in intracellular components and cell wall chemistry [27].

#### Effect of temperature on Pb(II) sequestration

To observe the effect of temperature on biosorption of Pb(II), time course studies were carried out with all the selected biosorbents at 25, 30, 40 and 50 °C (Table 3). It was observed that Pb(II) uptake usually improved by increasing temperature from 25 to 30 °C. The Pb(II) removal increases from 119.5 ± 0.6 to 174.2 ± 4.4, 49.1 ± 1.1 to 78.8 ± 1.3 and 110.5 ± 2.4 to 160.7 ± 1.3 mg/g at pH 4.5 with *A*. *caespitosus, A.* sp. RBSS-303 and *A. flavus* HF5 respectively by increasing temperature from 25 to 30 °C. Further increase in temperature from 30 to 50 °C resulted in increase in Pb(II) biosorption capacity from 174.2 ± 4.4 to 191.4 ± 3.5 mg/g dry weight with *A*. *caespitosus* but no significant increase (78.8 ± 1.3 to 82.4 ± 4.1 and 160.7 ± 1.3 to 168.3 ± 2.6 mg/g) was observed with other two biosorbents. Vijayaraghavan and Yun [28] justified that high temperature usually improves the adsorbate removal by increasing its kinetic energy and surface activity of biomass.

**Table 3:** Effect of temperature on Pb(II) biosorption capacity of screened fungal strains (W = 0.05 g, pH = 4.5, C_i_ = 100 mL, V = 100 mL, agitation rate = 180 rpm)

Biosorbents	Temperature (°C)			
	25	30	40	50
*A. caespitosus*	125.0 ± 2.6	174.2 ± 4.4	176.7 ± 2.3	183.6 ± 2.6
*A.* sp. RBSS-303	54.3 ± 2.6	79.9 ± 2.4	82.2 ± 4.9	93.3 ± 1.8
*A. flavus* HF5	110.6 ± 2.1	160.7 ± 1.7	164.9 ± 1.3	168.4 ± 2.4

The time course studies revealed that at 30 °C the equilibrium reached after about 6 h but on the other hand at 50 °C, the same percentage removal was attained very rapidly only after 0.5, 1 and 2 h of contact time with *A*. *caespitosus*, *A. flavus* HF5 and *A.* sp. RBSS-303 respectively. This uptake is in harmony with reported result that within 25 min of interaction 90% adsorption of cadmium is attained with dead biomass of marine algae *Fucus* sp. [29]. Similar results of rapid increase followed by a slower uptake rate was observed for biosorption kinetics of Ni(II) and Pb(II) by *Phanerochaete chrysosporium* and with seaweed biomass [29, 30].

#### Effect of biomass concentration on uptake capacities

**Fig. 2:**
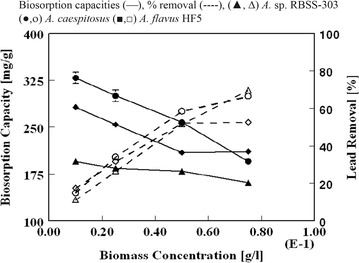
Pb(II) uptake capacities and  % removal by *A.* sp. RBSS-303, *A. caespitosus* and *A. flavus* HF5 as a function of biomass concentration (C_i_ = 200 mL, V = 100 mL, pH = 4.5, temperature = 28 ± 2 °C, agitation rate = 180 rpm, contact time = 6 h)

The effect of biosorbent dosage on Pb(II) biosorbing capacity and removal (%) from solution having 200 mg Pb(II)/dm^3^ for a period of 6 h incubation was also examined (Fig. 2). With raising biomass amount from 0.1 to 0.75 g/dm^3^ the maximum observed biosorption capacities (328.7 ± 8.8, 195.3 ± 2.1, and 282.0 ± 6.9 mg/g dry weight) were reduced to (195.3 ± 0.8, 161.1 ± 3.7, 212.9 ± 1.6 mg/g dry weight) for *A. caespitosus*, *A.* sp. RBSS-303 and *A. flavus* HF5 respectively. This decrease in sorption capacity at high biomass concentration would be a consequence of lesser availability of cell surface for metal binding due to cell aggregation [31]. However, in high concentration of biomass, there is a rapid superficial adsorption that produces a lower metal concentration in solution than the lower cell concentration. The extent of Pb(II) removal (%) from aqueous solution was found to increase with increase in cell concentration and percentage removal values of 66.5, 59.5 and 52.5% were attained for *A. caespitosus*, *A.* sp. RBSS-303 and *A. flavus* HF5 respectively at 0.75 g dry weight of biosorbent/dm^3^ of Pb(II) solution. Argun et al. [32] correlated the parallel tendency of metal removal efficiency and adsorbent dose with the increasing surface area of vacant binding sites.

#### Effect of initial Pb(II) concentration on uptake capacities

**Fig. 3:**
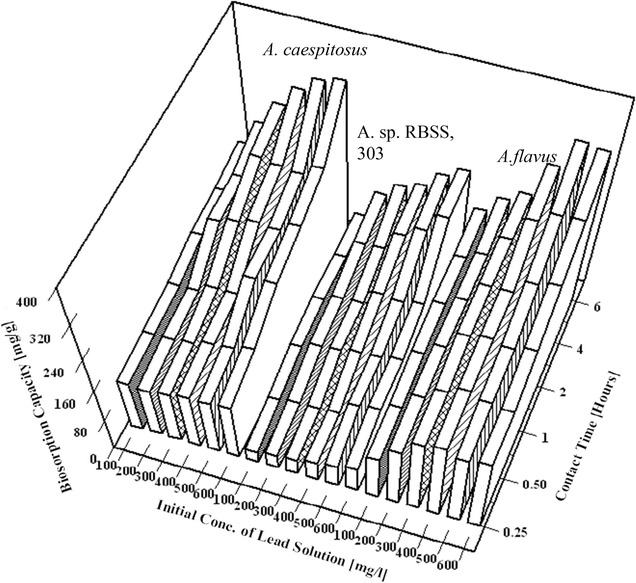
Time course studies on effect of initial Pb(II) concentrations on biosorption by *A. caespitosus*, *A.* sp. RBSS.303 and *A. flavus* HF5 (W = 0.05 g, V = 100 mL, agitation rate = 180 rpm, temperature = 28 ± 2 °C, pH = 4.5)

The effect of initial Pb(II) concentrations on biosorption capacity was studied by incubating 0.05 g biomass in 100 mL of Pb(II) solutions having 10–600 mg/dm^3^ concentration range using *A*. *caespitosus*, *Aureobasidium* sp. RBSS.303 and *A. flavus* HF5 as biosorbent (Fig. 3). The maximum examined biosorption capacity (q_max_) for *A. flavus* HF5 was 326.5 ± 3.1 mg/g at initial concentration of 500 mg Pb(II)/dm^3^ with no increase in uptake capacity with further increase in initial Pb(II) concentration up to 600 mg/dm^3^. At 500 mg/dm^3^ initial concentration of Pb(II) solution the observed biosorption capacities were 341.5 ± 7.1 mg/g by *A*. *caespitosus* and 214.8 ± 0.9 mg/g by *A.* sp. RBSS-303 that increased to 351.6 ± 5.7 and 235.1 ± 1.8 mg/g respectively at initial 600 mg/dm^3^ Pb(II) concentration. In the beginning adsorption rate is high as large available surface area is accessible to metal ions. With the passage of time the bare surface lessened rapidly with increasing coverage that gradually decreased the adsorption rate and leads to equilibrium. Thus, the initial Pb(II) concentration of 500 mg/dm^3^ showed the highest initial uptake. Rao et al. [33] reported that at higher cadmium ions concentrations (50–1000 mg/L), the uploading capacity of biosorbent increased from 2.23 to 25.64 mg/g due to increase of driving force i-e concentration gradient while the percentage removal decreased from 89.04 to 51.28% that was characterized as lack of active sites to accommodate more available metal ions in the solution.

The ratio of equilibrium concentration in solid and aqueous phase is distribution coefficient (D) (mg metal/mL solution), which can be calculated as:

8 $${\text{D}} = {\text{q}}_{\text{e}}/{\text{C}}_{\text{f}}$$

where, q_e_ = biosorption capacity at equilibrium (mg/g), *C*_f_ = Final concentration of sorbate (mg/mL).

**Fig. 4:**
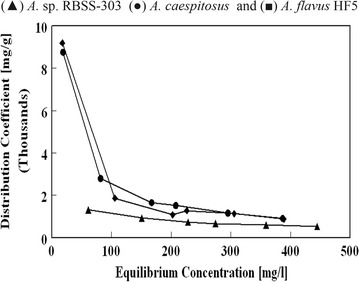
Distribution coefficient for Pb(II) biosorption by *A.* sp. RBSS-303, *A. caespitosus* and *A. flavus* HF5 (0.05 g dry weight/100 mL of Pb(II) solutions of various concentrations incubated for 6 h at 180 rpm and 28 ± 2 °C)

The high distribution coefficient (D) value of adsorption attribute to a good biosorbent. *A. caespitosus* and *A. flavus* HF5 exhibited maximum D values of 8749.11 and 8189.28 mL/g at C_e_ of 18.79 and 18.48 mg Pb(II)/dm^3^ respectively (Fig. 4). The loading capacity of *A.* sp. RBSS-303 was 1309.64 mL/g at C_e_ of 61.02 mg Pb(II)/dm^3^. At higher initial concentration of Pb(II) it decreased in the order of 908.8, 875.1 and 528.3 mL/g at C_e_ of 386.9, 389 and 444.9 mg Pb(II)/dm^3^ respectively with 0.5 g/dm^3^ biomass concentration. The lower values of distribution coefficient (D) with increasing Pb(II) concentrations (C_e_) results the low lead concentration in continuous aqueous phase than at sorbent–water interface. Akhtar et al. [27] also reported the value of distribution coefficient 3968 mL/g dry weight at *C*_e_ of 25 mg Zr/dm^3^ that decreased to 180 mL/g at C_e_ of 995 mg Zr/dm^3^.

### Equilibrium and kinetic studies

#### Equilibrium studies

**Fig. 5:**
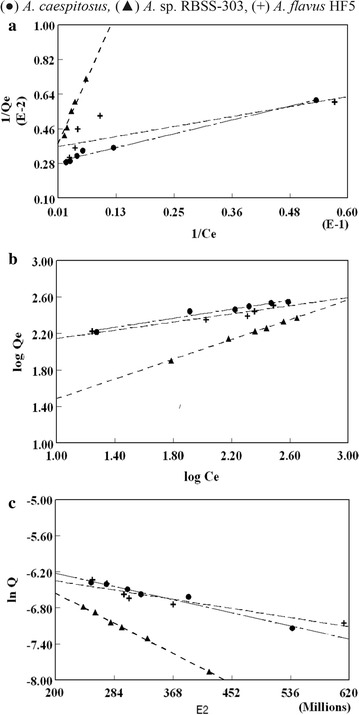
Langmuir (**a**), Freundlich (**b**) and Dubinin–Radushkevich (**c**) adsorption isotherms plots of Pb(II) biosorption to *A. caespitosus, A.* sp. RBSS-303 and *A. flavus* HF5

The relation between amount of adsorbate molecule at constant temperature and its concentration in equilibrium is called isothermal modelling. Isothermal study is used to estimate the total amount of adsorbent required to adsorb the requisite amount of a adsorbate from the solution. In the present study the equilibrium data of Pb(II) biosorption at 30 °C was analyzed using Langmuir, Freundlich and Dubinin–Raduskevich isotherms at various initial concentrations (from 100 to 600 mg/dm^3^) using fungal biosorbents (Table 4). From Langmuir plots the calculated correlation coefficient values were 0.99, 0.99 and 0.76 for *A. caespitosus*, *A.* sp. RBSS-303 and *A. flavus* HF5 respectively (Fig. 5a) at various initial Pb(II) concentrations. The theoretical values of “q_max_” were in agreement with experimental values in case of *A. caespitosus*. While theoretical value of “q_max_” for *A.* sp. RBSS-303 was high and in case of *A. flavus* HF5 was low as compared to experimental “q_e_” values. However, the calculated correlation coefficients from Freundlich plots were 0.98, 0.99 and 0.96 for *A. caespitosus*, *A.* sp. RBSS-303 and *A. flavus* HF5 respectively (Fig. 5b). The maximum sorption capacity values calculated by Freundlich isotherm were in harmony with experimental sorption capacity in case of *A. flavus* HF5 and *A.* sp. RBSS-303. The maximum value of q_exp_ of Pb(II) sorption on *A. caespitosus* was concordant to the calculated values using Langmuir model. While with *A.* sp RBSS-303 and *A. flavus* HF5, the sorption capacity (q_max_) values calculated from Langmuir isotherm were found to be deviated by 30.2 and 20.5% respectively when compared to that of experimental values (Table 4). The Langmuir model (r^2^ = 0.97) fits better than Freundlich (r^2^ = 0.8) for Pb(II) adsorption onto bael leaves [34]. The copper biosorption by brown alga *Fucus serratus* gives a improved description of investigated results with the Langmuir isotherm than the Freundlich equation [35].

**Table 4:** Comparison of q_max_ obtained from adsorption isotherms for Pb(II) biosorption

Biomass	Langmuir			Freundlich			Dubinin–Radusskevic		
	q_e_ (mg/g)	q_max_	b	R_L_	q_max_	K_F_	n (ml/mg)	q_max_	E_s_ (J/mol)
*A. caespitosus*	351.2	351.0	4.6 × 10^−2^	0.17	403.5	87.4	4.1	689.1	38.6
*A.* sp. RBSS-303	235.1	307.0	5.7 × 10^−2^	0.15	271.5	88.2	1.8	973.8	26.1
*A. flavus* HF5	346.6	274.8	8.2 × 10^−2^	0.11	346.3	83.7	4.5	522.4	16.6

*Es* sorption energy, *q*_*e*_ equilibrium sorption capacity, *q*_*max*_ maximum sorption capacity, *b* Langmuir constant, *K*_*F*_ adsorption capacity, *n* adsorption intensity

The dimensionless equilibrium parameter (R_L_) values, if lies greater than 0 and lesser than 1 indicate the favorable biosorption process [36]. Present studies (Table 4) proved the favorable Pb(II) biosorption with all used biosorbents. Ashraf et al. [37] also reported the favorable sorption of lead, copper, zinc and nickel on the biosorbent *Mangifer aindica* as separation factor values lies between zero and one.

The graph between surface coverage values (θ) and Pb(II) concentration demonstrate the direct relationship in initial metal ions concentration and biomass surface coverage until the surface is saturated. The surface coverage values for Pb(II) on absorbents are in the order of *A.* sp. RBSS-303 > *A. flavus* HF5 = *A. caespitosus.* The surface coverage values were approaching unity with increasing solution concentration indicating effectiveness of Ni(II) biosorption by *Cassia fistula* [38].

*Aureobasidium* sp. RBSS-303 and *A. flavus* HF5 for Pb(II) sorption have close Freundlich q_max_ with the experimental q_e_ with high correlation coefficient values. The Freundlich parameters K_F_ (relative sorption capacity) and 1/n specify whether the sorption nature is favorable or unfavorable [39]. The values of these constants (K_F_, n) are enlisted in Table 2 for Pb(II) sorption onto *A.* sp. RBSS-303 and *A. flavus* HF5. These results predict that Freundlich isotherm is followed by the sorption data very well with the interpretation that biosorbents possess heterogeneous surface with identical adsorption energy in all sites and the adsorbed metal ion interacts only with the active sites but not with others. However, this interpretation should be reviewed with caution, as the biosorption and isotherm exhibit an irregular pattern.

The q_max_ values computed from D–R isotherm were far away from the experimental q_e_ value for all the biosorbents for Pb(II) (Table 4), except *A. oryzae* SV/09 that have close q_max_ obtained from D–R isotherm with q_e_ but at the same time the correlation coefficient value is very low i.e. 0.80. Moreover, the straight lines obtained also not passed through the origin that is basic requirement of this model. The D–R adsorption isotherms for Pb(II) biosorption showed highest value of correlation coefficients 0.99 for *A.* sp. RBSS-303 however, for *A. caespitosus*, and *A. flavus* HF5 these values were 0.98 and 0.94 respectively (Fig. 5c).

#### Kinetic studies

**Fig. 6:**
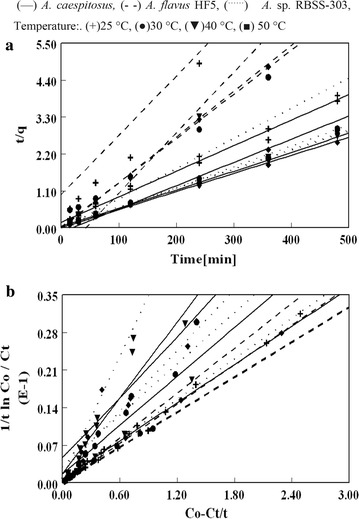
**a** Pseudo-second-order and **b** saturation mixed order kinetic plots of Pb(II) biosorption by *A. caespitosus*, *A.* sp. RBSS-303 and *A. flavus* HF5

Kinetics of Pb(II) adsorption on biosorbents were studied at temperature 25, 30, 40 and 50 °C with initial solution concentration of 100 mg/dm^3^. The experimental data for Pb(II) biosorption fits very well to pseudo-second-order kinetic models with highest correlation coefficient (0.99) using all biosorbents (Fig. 6). In recent years, the pseudo-second-order rate expression has been widely applied to the adsorption of pollutants from aqueous solutions.

The pseudo-second-order equation can be expressed as

9 $${\text{dq}}_{t} /{\text{d}}_{t} = {\text{k}}_{ 2} ({\text{q}}_{\text{e}} - {\text{q}}_{\text{t}} )^{ 2}$$

Integrating and applying the boundary conditions leads to

10 $$( {\text{t}}/{\text{qt}}) \, = { 1}/{\text{k}}_{ 2} {\text{q}}_{\text{e}}^{ 2} + \, \left( { 1/{\text{q}}_{\text{e}} } \right){\text{ t}}$$

Sorbed Pb(II) ions at equilibrium and time t are represented as q_e_ and q_t_ respectively. The values of k_2_, rate constant of pseudo-second-order (g/mg min) and q_e_, adsorption capacity at equilibrium (mg/g) were calculated from the slope and intercept of straight lines, obtained by plotting t/q_t_ against t for Pb(II) biosorption (Table 5). The theoretical values of “q_e_” obtained from pseudo-second-order expression were in good agreement with experimental “q_e_” values at all temperatures, proved the efficient application of these biosorbents in aqueous stream even at very low initial concentration of solute. Azizan [40] also reported that the adsorption process follow pseudo-second-order expression when the initial concentration of solute is low in solution. The pseudo-second-order expression in this studies also verify the mechanism of adsorption involving valency forces through the sharing or exchange of electrons between the adsorbent and adsorbate as covalent forces, and ion exchange. Adsorption which follow chemisorption gave pseudo second-order rate expression [41]. A number of other metal-biomass system in literature followed the pseudo second order kinetic [41–44].

**Table 5:** Evaluation of pseudo-second-order and saturation mixed order kinetic models, rate constants and q_e_ estimated for Pb(II) biosorption (C_i_ = 100 mg/L, pH = 4.5, biosorbent (dry weight) = 0.5 g/L, agitation rate = 180 rpm, temperature = 28 ± 2 °C)

Biosorbents	Temperature (°C)			
	25	30	40	50
*A. caespitosus*				
q_e, expt_	125.0 ± 2.6	174.2 ± 4.4	176.7 ± 2.3	183.6 ± 2.6
Pseudo-second-order				
qe	131.57	186.21	169.49	188.67
K_2_	3.5 × 10^−4^	3.9 × 10^−4^	− 8.8 × 10^−4^	1.4 × 10^−2^
*A.* sp. RBSS-303				
q_e, expt_	54.3 ± 2.6	79.9 ± 2.4	82.2 ± 4.9	93.3 ± 1.8
Pseudo-second-order				
qe	56.6	75.9	75.5	82.3
K_2_	3.1 × 10^−4^	1.3 × 10^−4^	− 4.3 × 10^−2^	−2.2 × 10^−2^
*A. flavus* HF5				
q_e, expt_	110.6 ± 2.1	160.7 ± 1.7	164.9 ± 1.3	168.4 ± 2.4
Pseudo-second-order				
qe	111.0	160.8	171.9	177.6
K_2_	2.7 × 10^−3^	8.4 × 10^−4^	−3.2 × 10^−3^	4.9 × 10^−4^

*q*_*e*_ equilibrium sorption capacity, *q*_*e, expt*_ experimental sorption capacity, *K*_*2*_ second-order rate constant, *R*_*val*_ correlation coefficient

### Thermodynamic studies

**Fig. 7:**
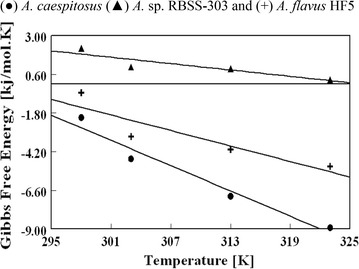
Thermodynamic studies of Pb(II) biosorption by *A. caespitosus*, *A.* sp. RBSS-303 and *A. flavus* HF5 at different temperatures

The feasibility and spontaneity of biosorption process is examined by thermodynamic study. Thermodynamic parameters characterize the biosorbent to calculate the free energy change (ΔG°) that deals with the viability of a reaction. The Gibbs parameter (ΔG°) is related to the standard thermodynamic equilibrium constant (K_D_°) of the biosorption system by the classic equation [45]:

11 $$\Delta {\text{G}}^\circ = \, - {{\text{RT ln K}}_{\text{D}}}^\circ$$

where, ΔG° is standard free energy change, R is universal gas constant, T is temperature in Kelvin. From the thermodynamics, K_D_° can be equal to apparent equilibrium constant (K_D_) at infinite dilute condition. Therefore, K_D_ can be obtained by calculating K_D_ at a different temperature and initial metal concentration and extrapolating to zero. Also, if the reasonable fit obtained with the Langmuir isotherm, the Langmuir equation constants can be used to calculate the Gibbs free energy change by the following equation:

12 $$\Delta {\text{G}}^\circ = \, - {\text{RT ln }}\left( {{\text{q}}_{\text{e}} .{\text{b M}}/{\text{V}}} \right)$$

where q_e_ (mg/g), and b (L/mg) are the Langmuir isotherm constants and M/V (g/L) is the biomass dosage, which make the product of q_e_·b as a dimensionless expression. The ΔG° for the biosorption of lead ions concentration 100 mg/dm^3^ in separate set of experiments was found at different temperatures (25, 30, 40 and 50 °C) using Langmuir isotherm constants. As shown in Fig. 7 ΔG° values decreased from − 2.0 to − 9.0 kJ/mole with *A. caespitosus*, from 2.2 to 0.3 kJ/mole using *Aureobasidium* sp. and from − 0.5 to − 5.1 kJ/mole with *A. flavus* HF5 in going from 298 K (25 °C) to 323 K (50 °C). At all investigated temperatures, biosorbents *A. caespitosus* and *A. flavus* HF5 have negative ∆G° values indicated the spontaneity of the process and also the mechanism of physical adsorption of Pb(II). Crini and Badot [46] removed dye from aqueous solution using natural polysaccharide in batch studies also concluded that the free energy of the process at all temperatures was negative and increased with the rise in temperature. In case of *Aureobasidium* sp. the positive value of ∆G° describe the non-spontaneous nature of the adsorption processes at the studied range of temperature. From slope and intercept of the plot between ΔG° vs T, the values of ΔS° (change in entropy) and ΔH° (change in enthalpy) were calculated by following equation [45].

13 $$\Delta {\text{G}}^\circ = \, \Delta {\text{H}}^\circ - {\text{ T}}\Delta {\text{S}}^\circ$$

ΔS° recommends the randomness either increasing or decreasing at the solid/solution interface in the system and ΔH° shows the route of energy in the system. The values of ΔS° and ΔH° for *A. caespitosus*, *A.* sp. RBSS-303 and A*. flavus* HF5 were found to be 26, 7, 16 J/mole K and 75, 21 and 46 kJ/mole respectively. The positive value of ΔS° revealed increase in disorderness of the system and decreasing trend at high temperature causing a change in biomass structure during the sorption process. The positive value of ΔH° indicated that an increase in the temperature is inclined with increase in adsorption capacity.

### Regeneration of biosorbents

#### Screening of superb eluent for metal elution

**Fig. 8:**
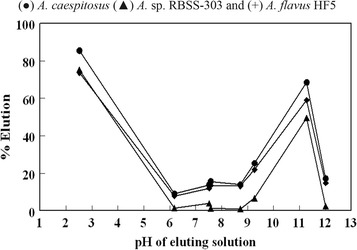
Effect of pH of desorbing agents on elution of lead from Pb(II) loaded biomass *A.* sp. RBSS-303, *A. caespitosus* and *A. flavus* HF5 (0.025 g dry weight of metal laden biomass was shaken for 1 h with 30 mL of eluent at 180 rpm and 28 ± 2 °C)

To study the pragmatic approach of biosorption function in treatment of industrial effluents, recoveries of adsorbed metal ions along with adsorbents reuse are very important [22]. However, greater part of biosorption research focused only on the sorption capacity of biosorbent with very little concern on regeneration of biomass [47]. This aspect was explored through Pb(II) desorption studies using a variety of desorbents [distilled water, HCl, CH_3_COONa, NaOH, Na_2_CO_3_, NaHCO_3_, (NH_4_)_2_SO_4_ and (NH_4_)_2_CO_3_]. Different desorbents were screened after 1 h contact time with Pb(II) loaded biosorbents during desorption studies. The initial pH of used desorbents (0.01 M) varied from 2.6 (hydrochloric acid) to 12.0 (sodium hydroxide). Plot of desorbents pH against desorption efficiency (Fig. 8) gave a liaison among pH and the respective efficiency values of desorbing agents. With HCl maximum desorption was observed to be 85.5, 75.3 and 73.7% from *A. caespitosus, A.* sp. RBSS-303 and *A. flavus* isolate HF5 respectively. Pandey et al. [48] suggested that in acidic pH adsorption was inhibited and efficient desorption of metal loaded biomass could be carried out. They found that inorganic acids (HCl, H_2_SO_4_ and HNO_3_) efficiently removed the Cd(II) loaded to the biomass. The elution efficiency decreased with increase in pH value 7.2 (H_2_O), little elution was observed. Elution efficiency again reached to the values 68.6, 49.6 and 59.1% at pH 11.3 (sodium carbonate) and dropped to 17.0, 2.4 and 14.8% at pH 12.0 (NaOH) for *A. caespitosus, A.* sp. RBSS-303 and *A. flavus* HF5 respectively.

#### Sorption/desorption cyclic studies

**Fig. 9:**
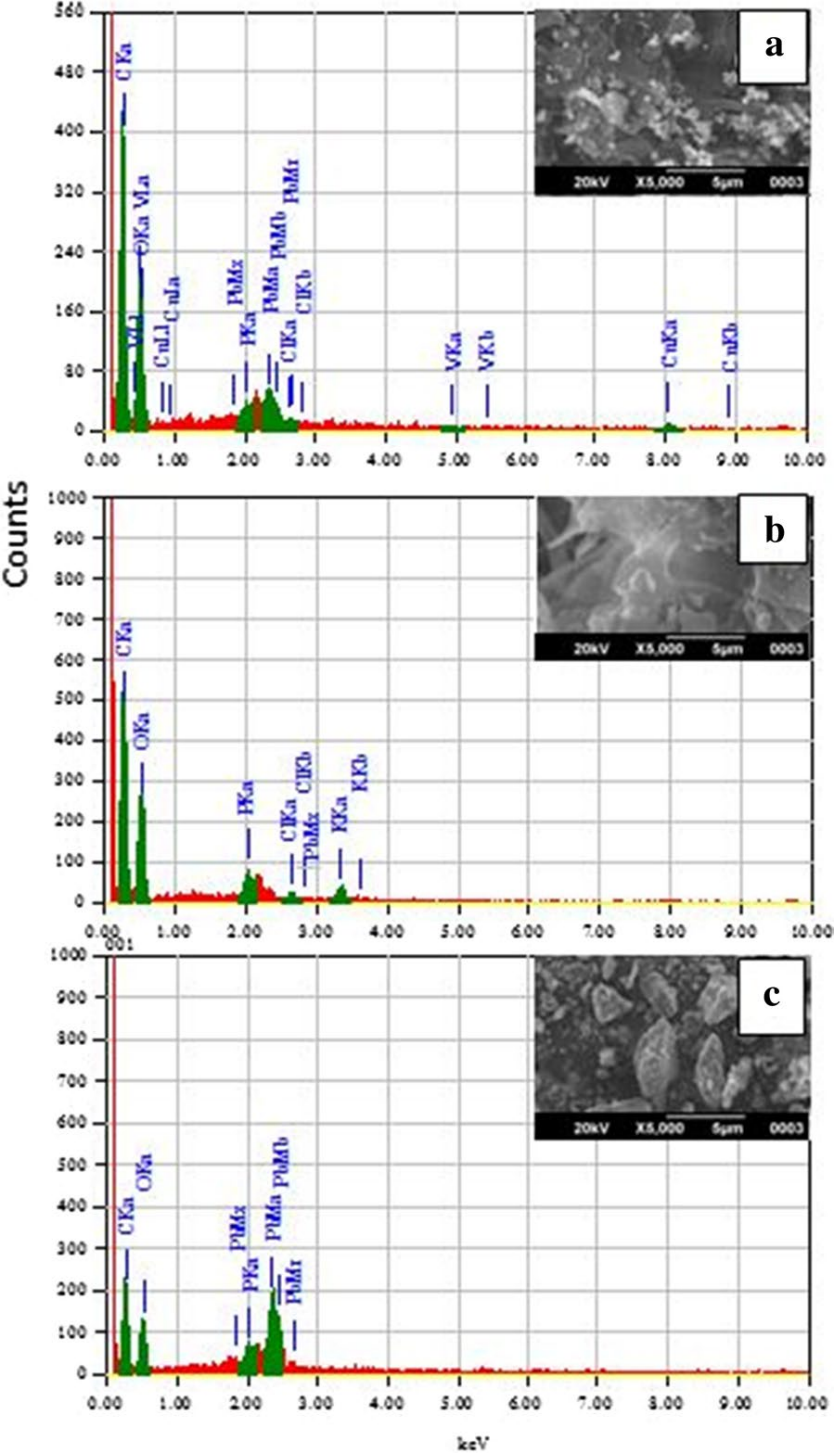
SEM-EDX micrograph of Pb(II) loaded biomass of *A. caespitosus* (**a**) *A.* sp. RBSS-303 (**b**), and *A. flavus* HF5 (**c**) after 6 h of exposure to 100 mg/L of Pb(NO_3_)_2_ solution at pH 4.5

For sorption/desorption cyclic studies, Pb(II) loaded biomass was generated after incubating 0.5 g biosorbent/dm^3^ of Pb(II) solution having initial concentration of 100 mg/dm^3^ for 6 h. The elution studies were carried out at optimized pulp density (0.83 g of metal loaded biomass per litre of 0.01 M HCl) after 2 h of incubation at 28 ± 2 °C and 180 rpm. The values of sorption capacity decreased from 174.2 to 152.9 and from 79.9 to 58.8 mg/g with *A. caespitosus* and *A.* sp. RBSS-303 and non-significant change (from 160.7 to 154.4 mg/g) was observed with *A. flavus* HF5 in going from first to fifth cyclic (Fig. 9). The decrease in uptake capacity in successive five cycles was *A.* sp. RBSS-303 > *A. caespitosus* > A*. flavus* HF5. The maximum decrease in % elution was experienced with *A.* sp. RBSS-303 while its value is almost same using *A. caespitosus* and *A. flavus* HF5 during five cycles (Table 6). The decline in metal sorption in frequent regeneration cycles was justified due to loss of active sites on biomass surface by acid treatment [49]. However, this drawback can be compensated by easy and massive growth of these fungal isolates, further optimization and immobilization.

**Table 6:** Percentage decrease in Pb(II) sorption capacities after five succeeding sorption–desorption cycles

Biosorbents	Sorption capacity (mg/g)		% decrease in sorption capacity
	1st	5th	
*A. caespitosus*	174.2	152.9	12.2
*A.* sp. RBSS-303	79.9	58.8	26.4
*A. flavus* HF5	160.7	154.4	3.9

### Scanning electron micrograph–energy-dispersive X-rays analysis (SEM–EDAX)

Scanning electron microscopy is a useful technique to study the morphology of biosorbent and its modification after sorbate interactions. SEM–EDX analysis of Pb(II) biosorption by dried powdered biomass after 6 h of contact time with 100 mg/L Pb(NO_3_)_2_ solution at pH 4.5 were shown in Fig. 9. SEM micrographs of powdered Pb(II) loaded fungal biomass showed the presence of dense metal crystals on the smooth surface of unloaded biosorbents. The spectra revealed the presence of C, O, P, Cl and K elements with no Pb(II) deposits in native biomass. While after Pb(II) exposure the spectra showed the major peaks of Pb(II) with 30.9, 14.2, 21.6 mass percent by replacing the peaks for K and Cl and decreased the mass percent of other elements (C, O, P). These findings facilitate to confirm that sorption, precipitation and ion exchange on the surface might be the major mechanisms for removal of Pb(II) from aqueous solution using fungal biomass. Kumar et al. [50] also characterized the biosorption of Pb(II) and Cu(II) from aqueous solutions onto Andean *Sacha inchi* shell biomass (SISB) by SEM that revealed the enhancement in weight percent of Pb(II) (15.72%) and Cu(II) (6.33%) ions on the surface of SISB after biosorption. Similarly Siddiquee et al. [51] also advocated the filamentous strains of fungi as future cleaning up microorganism and potential alternative to synthetic resins due to having versatile biosorption group; can grow and work under extreme conditions for remediation of dilute solution of metals and solid wastes.

## Conclusion

These studies demonstrated the comparative feasibility and selectivity of *A*. *caespitosus*, *A.* sp. RBSS-303 and *A. flavus* HF5 to remove Pb(II) from aqueous solution. The results endorsed that the sorption performance was strongly affected by culture age and physical state of fungal biomass, temperature, initial Pb(II) concentration and sorbent dosage. The kinetic studies showed the good fit of adsorption equilibrium data to pseudo-second order model. The high correlation coefficient values (0.99) of equilibrium experimental data proved the favorable biosorption process. Elution studies were carried out to regenerate the biomass using 0.01 N HCl with 85.5, 75.3 and 73.7% Pb(II) elution from *A. caespitosus; A.* sp. RBSS-303 and *A. flavus* isolate HF5 respectively. Finally, sorption/desorption cyclic studies indicated that *A. caespitosus, A.* sp. RBSS-303 and *A. flavus* HF5 could be used as cost effective, easily cultivatable future cleaning up microorganisms for removal and recovery of Pb(II) from polluted water stream.

## Abbreviations

b: Langmuir constant
C_des_: concentration of metal desorbed into the eluent (mg/L)
C_f_: final metal ion concentration (mg/L)
C_i_: initial metal ion concentration (mg/L)
E_s_: sorption energy (J/mole)
K: adsorption distribution coefficient. (mg metal/g biosorbent/mg ml)
k_2_: pseudo-second order rate constant
K_F_: Freundlich adsorption capacity
n: adsorption intensity
q: biosorption capacity (mg/g)
q_des_: eluted metal contents per gram of the biosorbent (mg/g)
q_e_: amount of biosorption at equilibrium (mg/g)
q_max_: maximum amount of metal ions per unit mass (mg/g)
q_t_: amount of biosorption at time ‘t’ (mg/g)
V: volume of metal ion solution (litre)
W: weight of biosorbent (g)
